# ImmunogenIcity of thE PandemRIX A (H1N1) 2009 Influenza vaccine in hemodialysed and renal transplanted patients

**DOI:** 10.1186/1753-6561-5-S1-P4

**Published:** 2011-01-10

**Authors:** A Hombrouck, N Broeders, L Labriola, D Abramowicz, M Jadoul, P Goubau, S Van Gucht, B Brochier, I Thomas

**Affiliations:** 1 National Influenza Centre, Communicable and Infectious Diseases, Scientific Institute of Public Health, Brussels, 1050, Belgium; 2 Erasmus hospital, Brussels, 1070, Belgium; 3 University hospital St. Luc, Brussels, 1200, Belgium

## 

Swine-origin pandemic human influenza A(H1N1)2009 rapidly spread around the world since its initial reporting on the 25^th^ of April 2009. To counter the virus, different vaccines were developed. In Belgium, the Pandemrix vaccine of GSK was used. Part of the Belgian population was vaccinated, especially those belonging to risk groups (immunodepressed patients, healthcare providers, patients with chronic disease, pregnant women….). In general, vaccine immunogenicity is less effective in immunodepressed patients than in immunocompetent patients.

In this study the immunogenicity of the Pandemrix vaccine was investigated by measuring neutralizing antibodies against A(H1N1)2009 in sera collected from immunocompetent and immunodepressed patients such as hemodialysed and renal transplanted patients. In total 32 healthy volunteers (control group), 106 hemodialysed patients and 112 renal transplanted patients were recruited. Neutralizing antibodies were measured in sera collected before (day 0) and 1 month after a single shot vaccination (day 30) using a seroneutralisation (SN) assay followed by an immunoenzyme colorimetric reaction to detect influenza A(H1N1)2009 antigen in infected cells. Geometric mean (GM) titers were determined at subject level by individual GM of quadruplicates at each time point. Individual and group level titer ratios (day 30/day 0) with 95% confidence intervals were determined as well.

The GM titer ratio for the healthy control group was 38 (23-62). For the dialysed patients, the GM titer ratio was 11 (8-17) and for the group of renal transplanted patients, the GM titer ratio was 5 (3-6). Differences in GM titer ratio between the healthy control group and each group of immunodepressed patients were significant (p<0,05; T test). Thirty out of 32 healthy participants (93%) had at least four-fold increases in SN titers between day 0 and day 30 (seroconversion). Among dialysed participants, 64 out of 106 (60%) had at least four-fold increases in SN titers between day 0 and day 30, and among renal transplanted patients, 49 out of 112 participants (44%) seroconverted between day 0 and day 30. Differences in seroconversion rates between the healthy control group and each group of immunodepressed patients were significant (p<0.05; fisher exact test).

These results suggest that only 60% of dialysed individuals developed sufficient neutralizing antibody titers after immunization with this vaccine. As for renal transplanted patients, only 44% of the patients developed sufficient neutralizing antibody titers after vaccination. Alternative vaccines, dosing, adjuvants or schedule strategies are needed to achieve effective immunization of these vulnerable populations.

